# The Effect of Selected Cathinones on Natural Cell Membranes: Microelectrophoretic Methods

**DOI:** 10.3390/molecules31020234

**Published:** 2026-01-09

**Authors:** Anna Trynda, Katarzyna Karwowska, Weronika Karpowicz, Katarzyna Kazimierska-Drobny, Aneta D. Petelska

**Affiliations:** 1 Faculty of Chemistry, University of Bialystok, Ciolkowskiego 1K, 15-245 Bialystok, Poland; a.trynda@uwb.edu.pl (A.T.); k.karwowska@uwb.edu.pl (K.K.); 2 Clinical Research Center, Medical University of Bialystok, M. Skłodowskiej-Curie 24a, 15-276 Bialystok, Poland; 3 Faculty of Mechatronics, Kazimierz Wielki University, Chodkiewicz 30, 85-867 Bydgoszcz, Poland; kkd@ukw.edu.pl

**Keywords:** cathinones, mephedrone, clephedrone, erythrocytes, thrombocytes, microelectrophoresis

## Abstract

Synthetic cathinones are cathinone analogues that humans have artificially created. The first compounds appeared on the European market in 2005. They belong to a class of drugs called stimulants, classified as new psychoactive substances. Synthetic cathinones are very popular; people use these drugs because they are cheaper “substitutes” for other stimulants. They produce psychostimulant and hallucinogenic effects similar to cocaine, amphetamine, and MDMA, among others. Despite their presence on the market for several years, the precise toxicological impacts of these compounds on the human body remain unknown. Studies were conducted on the effects of selected cathinones (mephedrone, clephedrone) on blood cells: erythrocytes and platelets. The effect of cathinones was determined by measuring the surface density of biological membranes using microelectrophoresis. The continued popularity of these compounds, coupled with limited knowledge of their precise effects on the human body, makes the problem significant and requires ongoing research. Based on the results obtained for mephedrone and clephedrone, it can be concluded that at the tested concentrations (170 ng/mL and 2700 ng/mL), they alter the surface charge density of the biological membranes of red blood cells and platelets.

## 1. Introduction

Cathinone (2-amino-1-phenylpropanone or a-aminopropiophenone) is a centuries-old alkaloid with psychoactive properties. It is found in the Khat bush, which is commonly grown in East Africa and the northeastern part of the Arabian Peninsula. It is used for mild intoxication in many African countries [1].

Interest in this substance in Europe grew at the start of the 21st century, coinciding with the emergence of NPS (New Psychoactive Substances). Since then, cathinones have been gaining popularity and have become an increasing social threat in Europe. Data from the European Union Drugs Agency (EUDA) show that the number of cathinone seizures, uncovered illegal laboratories producing cathinones, and their consumers are all growing [2].

In a survey conducted by the EUDA, 9% of respondents reported consuming cathinones in the last 12 months, and this number is still increasing [2]. For example, in Poland, cathinones constitute over 90% of identified new psychoactive substances [3].

The most popular cathinones include mephedrone (4-MMC), clephedrone (4-CMC), and clophedrone (3-CMC). New compounds continue to emerge. In 2022, EUDA issued a report on the risks associated with the use of 3-CMC [4]. Recently, work began to assess the risks posed by three additional synthetic cathinones seen in the European drug market: 2-methylmethcathinone (2-MMC), 4-bromomethcathinone (4-BMC), and N-ethylnorpentedrone (NEP) [5].

**Figure 1:**
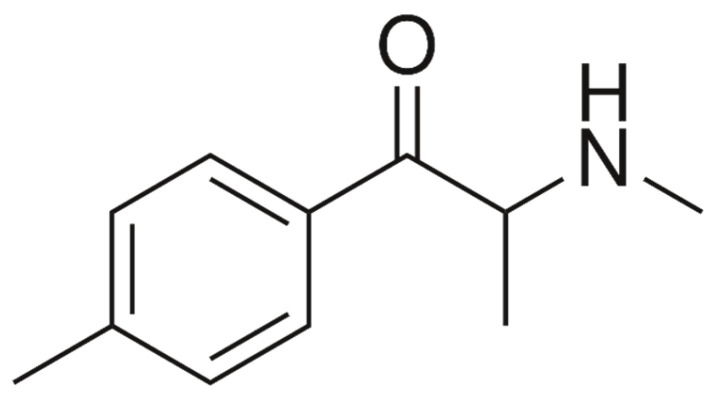
Structural formula of mephedrone.

Mephedrone (Figure 1), whose chemical name is (RS)-1-(4-methylphenyl)-2-methylaminopropan-1-one, is commonly referred to as 4-MMC or 4-methylmethcathinone. It is one of the most popular synthetic cathinones and has the molecular formula C_11_H_15_NO.

Mephedrone was first synthesized in 1929 [6,7,8,9] for therapeutic purposes [9]. It began to appear in Europe in 2007 as a recreational drug and has since attracted a broad audience [6,7,10]. Its popularity has contributed to numerous poisonings, including fatalities. Mephedrone acts as a central nervous system stimulant and empathogen, producing effects similar to amphetamine, cocaine, and MDMA [7,11,12]. It can be challenging to determine the dose responsible for specific effects or death [13,14].

**Figure 2:**
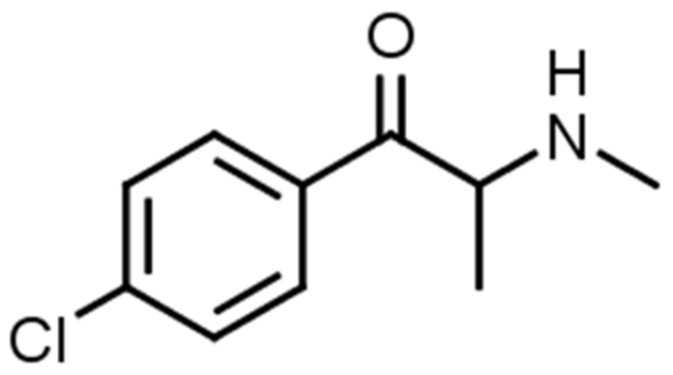
Structural formula of clephedrone.

Following the discussion of mephedrone, another notable cathinone derivative is clephedrone (1-(4-chlorophenyl)-2-(methylamino)propan-1-one, Figure 2). Also known as 4-chloromethcathinone or 4-CMC, it has the molecular formula C_10_H_12_ClNO [12,15,16].

Clephedrone appeared in Europe in 2014 [10,12,15,17] and reached Poland in the first half of 2015 [10]. It was identified as a successor to mephedrone and was placed under control in most European countries after 2015. In Poland, it was placed under legal control in 2018. In Poland, this compound remains popular and is among the most commonly identified substances in the synthetic cathinone group [18,19]. Unfortunately, its “fame” also has an adverse effect, as it is the compound most frequently identified in cases of fatal poisoning [20,21,22,23]. Although clephedrone has been available on the drug market for several years, there are still few detailed studies on the pharmacology of this substance [15,17]. However, its effects are considered similar to those of mephedrone [10,24] or amphetamine [25]. Current studies are mainly based on observations of mice [26] or rats [27] in laboratory conditions. Clephedrone has also been shown to have a strong addictive effect [12]. Despite many deaths caused by this substance, the lethal dose is still difficult to estimate [24].

Illegal production of synthetic cathinones is a major concern in Poland, which has become one of the leading producers in Europe. The size and scale of laboratories producing cathinones illegally vary: from small, “kitchen labs” producing several to a dozen or so grams in a single synthesis run, to large, almost industrial laboratories capable of producing 100 or more kg of finished product in a single synthesis run [5,10,28]. Production is a relatively simple process and involves two stages: bromination and amination of the bromine derivative. In practice, most illegal laboratories conduct synthesis in a single stage, starting with the bromine derivative, which is then aminated. The reason for this is the easy availability of the substrate—the bromine derivative and its low price, which significantly speeds and simplifies the entire process. For this reason, developing new solutions for detecting cathinones is extremely important.

This paper describes research on the effects of selected cathinones on blood cell counts. The study examined erythrocytes and thrombocytes. The impact of cathinones (mephedrone, clephedrone) was determined by measuring the surface charge density or zeta potential of the blood cell counts. Zeta potential is a measure of electrostatic interactions between particles and can be used to assess dispersion stability. For electrostatically stabilized systems, the higher the zeta potential, the more likely the dispersion will be stable. In aqueous systems, a value of approximately ±30 mV is considered the threshold for system stability. A biological membrane is a fundamental requirement for the existence of every cell. All biochemical processes occurring in the environment surrounding the membrane and within it influence proper cell function. The surface charge of a membrane reflects the membrane’s equilibrium, and many factors, such as membrane composition, environment, and solution pH, influence its value.

Synthetic cathinones were selected due to their widespread use, the rapid growth in consumption, numerous seizures, and the increasing number of illegal laboratories producing these compounds, compared to other groups of new psychoactive substances. The continued popularity of these compounds and the limited understanding of their precise effects on the human body make this issue significant and warrant ongoing research.

## 2. Results and Discussion

The natural cell membrane is a bilayer system: the inner layer mainly contains negatively charged phosphatidylserine, while the outer layer comprises neutral phospholipids, such as DPPC and sphingomyelin [29]. Phospholipids acquire specific charges at suitable pH values. To study electrostatic interactions between the membrane and other molecules, microelectrophoresis was used [30,31]. This method determines the cell membrane’s surface charge density (zeta potential) by measuring electrophoretic mobility. Understanding surface charge density is crucial for detecting how substances affect membrane charge.

Generally, at all physiological pH values, the net membrane charge is negative, ranging from several to several tens of µCm^−2^, and biological membranes have an isoelectric point around pH 4 [32,33]. The surface charge density and zeta potential of the cell membrane are important parameters that characterize its equilibrium state. These parameters strongly depend on environmental conditions, such as pH and membrane composition. Changes in the surface charge density of the cell membrane are observed in vitro: the addition of ionizable surfactants [34], as well as in vivo during lethal carbon monoxide poisoning [35].

**Figure 3:**
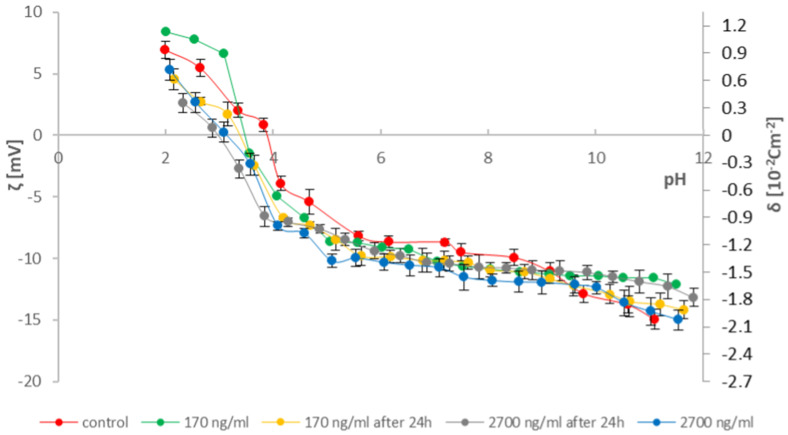
Dependence of zeta potential and surface charge density on the electrolyte pH of erythrocyte membranes modified with mephedrone at doses of 170 ng/mL and 2700 ng/mL immediately after isolation from whole blood and after 24 h.

**Figure 4:**
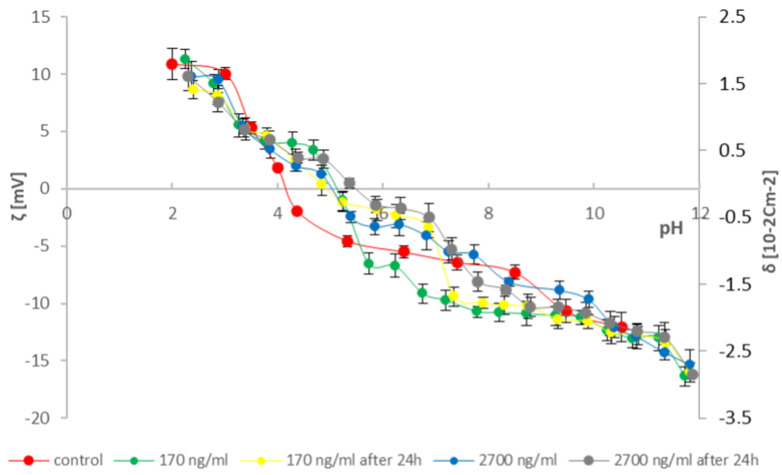
Dependence of zeta potential and surface charge density on the electrolyte pH of thrombocyte membranes modified with mephedrone at doses of 170 ng/mL and 2700 ng/mL immediately after isolation from whole blood and after 24 h.

**Figure 5:**
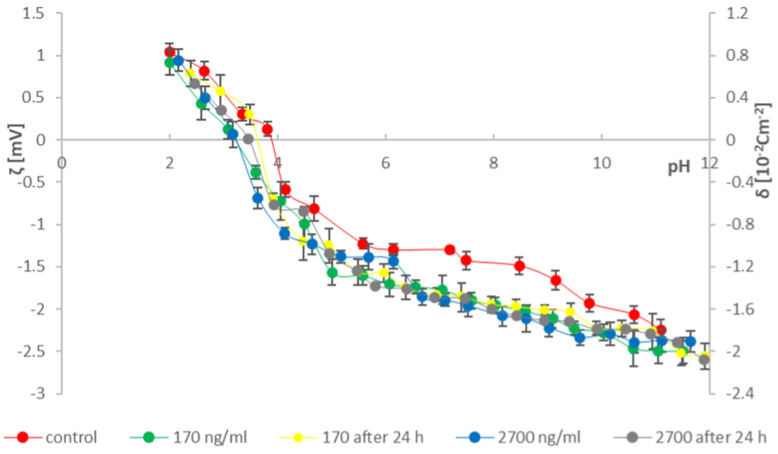
Dependence of zeta potential and surface charge density on the electrolyte pH of erythrocyte membranes modified with clephedrone at doses of 170 ng/mL and 2700 ng/mL immediately after isolation from whole blood and after 24 h.

**Figure 6:**
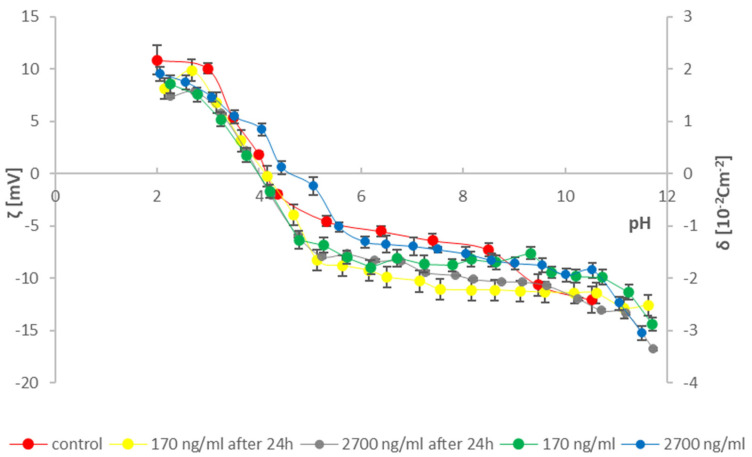
Dependence of zeta potential and surface charge density on the electrolyte pH of thrombocyte membranes modified with clephedrone at doses of 170 ng/mL and 2700 ng/mL immediately after isolation from whole blood and after 24 h.

To provide as much information as possible on the effects of cathinones on natural lipid membranes, studies were conducted on erythrocytes and thrombocytes isolated from blood. According to the literature data, two concentrations of cathinones were adopted [36]: 170 ng/mL—in cases of poisoning where death did not occur (range of possible concentrations in the human body 13–412 ng/mL) and 2700 ng/mL (range of possible concentrations in the human body 51–22,000 ng/mL)—in fatal cases. Measured solutions were prepared by adding 170 and 2700 ng/mL of the tested modifying substances (mephedrone and clephedrone) to the blood morphotic elements samples after isolation and 24 h after isolation from whole blood. The research used the method of microelectrophoresis, which indirectly enables the determination of the electric charge on the surface of the membrane (by measuring the electrophoretic mobility). Measurements were made on the dependence of the surface charge density of the blood morphotic elements membrane as a function of the pH of the electrolyte (range 2–10) (Figure 3, Figure 4, Figure 5 and Figure 6). The results are presented in Table 1, Table 2, Table 3 and Table 4.

Based on the curves in Figure 3 and the data in Table 1, it can be observed that at low pH values, the addition of mephedrone increases the positive zeta potential (surface charge density). In contrast, at a concentration of 2700 ng/mL, the addition of mephedrone causes a decrease in the positive zeta potential (surface charge density) of erythrocyte membranes compared to the control curve. Furthermore, in the curves obtained 24 h after the addition of mephedrone to whole blood, a decrease in the positive surface charge density of erythrocyte membranes is observed at both concentrations. As pH increases, a slight deviation in zeta potential is observed at both concentrations, with a trend toward more negative values than in the control curve. Notably, the results at both tested concentrations (170 and 2700 ng/mL) indicate similar surface charge densities at higher pH values. Additionally, for all samples containing mephedrone, the isoelectric point shifts toward lower pH values.

Based on the curves in Figure 4 and the data in Table 2, a slight decrease in the positive zeta potential (surface charge density) of platelet cell membranes is observed across all relationships compared to the control curve in the pH range of 2–4. Subsequently, an increase in the surface charge density of platelet membranes is observed up to pH 5.5. Around pH 6, an increase in the negative surface charge density is observed only for the platelet sample spiked with mephedrone at 170 ng/mL, isolated directly after the addition of cathinone. In contrast, for the remaining samples, a higher negative surface charge density is observed compared to the control curve. A shift in the isoelectric points toward higher pH values is also observed compared to the control group.

Based on the curves presented in Figure 5 and the data in Table 3, a decrease in positive and an increase in negative values of the surface charge density of red blood cell membranes is observed for all analyzed samples compared to the control curve. The zeta potential values obtained for the analyzed erythrocyte membrane samples with clephedrone are similar across the entire pH range. A slight shift in the isoelectric points towards lower pH values is also observed.

Based on the curves in Figure 6 and the data in Table 4, a decrease in positive zeta potential and an increase in negative zeta potential are observed in the pH range 2–9 compared to the control. This is followed by a decrease in negative zeta potential values of platelet membranes compared to the control. A different pattern appears for the sample with 2700 ng/mL clephedrone added directly to isolated platelets in the pH range 3–6. For this sample, only a shift in the isoelectric point compared to the control is observed.

However, during this period, scientists, mainly through observations of clinical and addicted patients, discovered the effects of cathinones on humans [37,38,39]. No studies on the effect of cathinones on the estimation of the surface charge density of morphotic elements in human blood were conducted.

Analyzing the obtained curves and the information in the tables reveals differences both between the tested compounds and within the studied groups. Analyzing the results for individual groups, the curves differ depending on the sample preparation method. In erythrocytes modified with mephedrone, a difference is noticeable in the shift in the surface charge density at low pH values and in the position of the isoelectric point at a concentration of 170 ng/mL. In the case of thrombocytes, the curves are very similar, whereas in samples isolated at a concentration of 170 ng/mL, an increase in the surface negative charge density is observed compared to the curve analyzed after 24 h. The results obtained for erythrocytes modified with clephedrone differ slightly. Differences in the curves are observed in the tested thrombocytes, particularly at 2700 ng/mL.

Synthetic cathinones have effects similar to those of known stimulants, such as MDMA, amphetamine, and cocaine [38,39,40,41,42,43,44,45]. Like other stimulants, they affect the central nervous system (CNS), but with a lower intensity than their corresponding phenylethylamine analogues. This lower potency is due to the β-keto group, which makes the molecule more polar and less able to cross the blood–brain barrier [38]. The action of synthetic cathinones is based on the inhibition of SLC6 monoamine transporters, such as the serotonin (SERT), dopamine (DAT), and noradrenaline (NAT) transporters [45]. Interaction with monoamines is the primary mode of action of cathinones. Regardless of the molecular mechanism, all synthetic cathinones increase extracellular monoamine concentrations in the brain, thus amplifying cell-to-cell signaling [46].

Depending on the compound ingested and its dose, the effects may vary in intensity. The individual’s predispositions also play a role [35]. In our study, we used two doses of cathinones: a non-lethal dose (170 ng/mL) and a lethal dose (2700 ng/mL). Changes in zeta potential and surface charge density values can be observed. This is likely related to changes in the levels of all membrane components (phospholipids and integral membrane proteins) in erythrocytes and platelets.

Determining the appropriate relationship between a substance’s dose and its health consequences is problematic. This may be due to the instability of cathinones in biological matrices. Their concentration at the time of sample collection may differ from that at the time of analysis. The difference depends on the storage method and the time after which the sample was analyzed [13,14,44]. Cathinones are metabolized in the liver [47]. Almost all cathinones are excreted in urine unchanged [11,48].

Cathinones are typically characterized by a short duration of action—2 to 4 h when taken orally [40]. The typical dose of the most common cathinone, mephedrone, is 100–250 mg. Because its effects are short-lived, users may take several doses in a row (up to 1 g per session), which can easily lead to overdose [21,37]. Our results confirm the short duration of action. Tests on samples prepared both immediately after isolating morphotic blood elements and on samples isolated 24 h after cathinone addition (with 170 or 2700 ng/mL of mephedrone or clephedrone) show similar zeta potential values.

To our knowledge, this is the first report to describe the effect of cathinones on cell membrane surface properties. However, our study is a preliminary in vitro study, and the results cannot be directly extrapolated to in vivo effects. More in-depth research will provide essential insights into biological phenomena.

## 3. Materials and Methods

### 3.1. Materials

*Reagents:* Mephedrone (C_11_H_15_NO∙HCl, 99.5%, LoGiCal, Lodz, Poland), clephedrone (C_10_H_12_ClNO·HCl, 99%, Biotechnology Park, Lodz, Poland) were purchased and used without any further purification. The molecular weights of the materials used were approximately 213.7 and 234.1 g·mol^−1^ for mephedrone and clephedrone, respectively. These reagents were purchased for research and are strictly accountable materials, and the quantities entered are recorded in a register and stored in a safe.

Sodium chloride, hydrochloric acid, and sodium hydroxide (pure, Avantor Performance Materials Poland S.A., Gliwice, Poland) were used to prepare electrolyte solutions.

*Isolation of erythrocytes from whole blood:* To isolate red blood cells, whole blood must be centrifuged appropriately. Blood from the Blood Donation Center in Białystok was used for the study (the Ethics Review Board of the Medical University of Bialystok approved the study, No. R-I-002/533/2010). The collected blood was placed in sterile containers and sent to the Faculty of Chemistry at the University of Bialystok for further research. 7 mL of whole blood was measured in a test tube and centrifuged in an MPW-350 centrifuge (MPW MED. INSTRUMENTS, Warsaw, Poland) for 8 min at 900 rpm. The centrifugation yielded platelet-rich plasma, a buffy coat layer, and a pellet containing red blood cells. The platelet-rich plasma supernatant was carefully collected from the pellet and transferred to a separate tube for storage until the next step. The buffy coat fraction above the red blood cell layer was removed and discarded. The remaining red blood cell fraction was washed with isotonic saline (0.9% NaCl solution) by centrifugation at 3000 rpm. Three washing cycles were performed. This procedure was intended to eliminate plasma proteins, as well as any leukocytes, platelets, and microaggregates. The purified erythrocytes were suspended in a 0.9% NaCl solution and retained for further study [31].

*Isolation of thrombocytes from whole blood:* The platelet-rich plasma obtained in the previous step was centrifuged for 8 min at 4000 rpm. Centrifugation yielded platelet-poor plasma and a white platelet precipitate layer. The resulting plasma was collected from the precipitate and discarded. The platelet precipitate, like the erythrocytes, was washed three times with physiological saline solution and centrifuged for 15 min at 3000 rpm. The obtained platelets were suspended in a 0.9% NaCl solution and retained for further analysis [31].

*Electrolyte solutions:* The electrolyte solutions (0.155 M NaCl) were prepared using ultrapure water. The water was obtained by the Milli-Q plus water purification system (resistivity of 18.2 MΩ cm) from Millipore, Burlington, MA, USA.

The solutions used in the tests were

- 0.155 M NaCl solution obtained by dissolving 9 g of NaCl in 1 L of ultrapure water.
- 0.1 M HCl solution with added NaCl obtained by dissolving 4.18 mL of 36% HCl and 1.58 g of NaCl in 500 mL of ultrapure water.
- 0.1 M NaOH solution with added NaCl obtained by dissolving 2 g of NaOH and 1.58 g of NaCl in 500 mL of ultrapure water.

### 3.2. Sample Preparation

Samples were prepared in two ways. First, appropriately measured amounts of substances were added to previously isolated erythrocytes and platelets to obtain concentrations of 170 ng/mL and 2700 ng/mL, respectively. The prepared samples were then subjected to electrophoretic mobility measurements using a Zetasizer Nano ZS at various pH values. Second, the substances were added directly to whole blood to obtain concentrations of 170 ng/mL and 2700 ng/mL. These samples were incubated for 24 h at 4 °C in a refrigerator. After this period, red blood cells and platelets were isolated and subjected to electrophoretic mobility measurements using a Zetasizer Nano ZS as a function of pH in the electrolyte solution [31].

### 3.3. Microelectrophoretic Measurements

**Figure 7:**
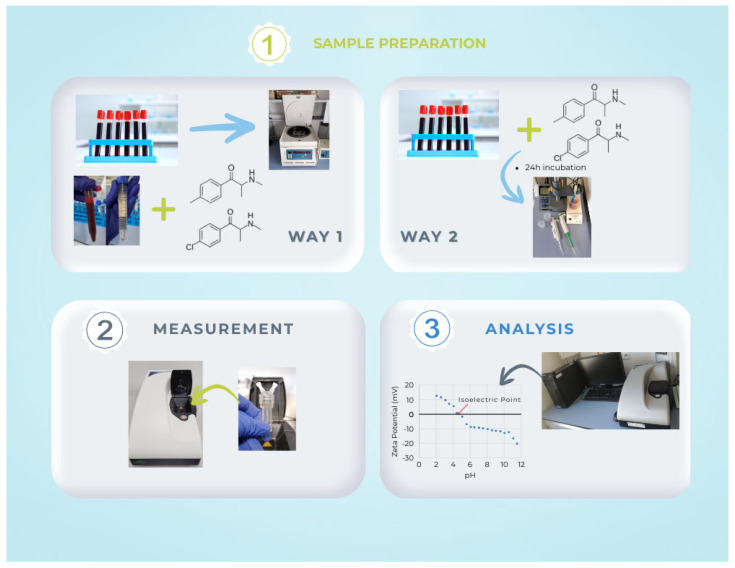
Schematic diagram of the experiments performed.

The Zetasizer Nano ZS apparatus (Malvern Instruments, Malvern, UK) was used to perform microelectrophoretic measurements. A laboratory meter, WTW InoLab pH 720 (WTW, Weinheim, Germany), was used to measure pH. Measurements of electrophoretic mobility in pH 2–9 were made. A voltage applied to the cell electrodes creates a uniform electric field. This causes charged particles to move towards the appropriate electrode. Their charge determines the direction of particle movement. The analyzer uses LDV (Laser Doppler Velocimetry) methods to measure particle velocity via the Doppler effect. Figure 7 shows a diagram of the experiments performed.

The velocity of the particles is measured and expressed as electrophoretic mobility [31,35,49,50]. Six measurements of electrophoretic mobility were made for each pH value of the analyzed samples (each covering 100–200 series, duration 5 s). All experiments were repeated three times. The summarized results are expressed as mean values with the determined standard deviation. The results were analyzed using standard statistical methods, and the error bars are shown in Figure 3, Figure 4, Figure 5 and Figure 6. The data also were analyzed by the ANOVA test for comparisons between control and samples with catinones. Values of *p* ≤ 0.05 were considered significant.

### 3.4. Determination of the Surface Charge Density of Cell Membranes by Measuring Electrophoretic Mobility

The studies performed utilized microelectrophoresis, which allows for indirect determination of the electric charge on the membrane surface through measurement of electrophoretic mobility. The surface charge density describes surface charge. Using the experimental data of electrophoretic mobility, the value of the surface density was determined using Equation (1):

(1) $$\delta =\frac{\eta \cdot u}{d}$$

where η is the viscosity of the solution, u is the electrophoretic mobility, and d is the thickness of the diffuse layer [51].

Using Equation (2), the thickness of the diffusion layer was determined:

(2) $$d=\sqrt{\frac{{\varepsilon \varepsilon }^{0}RT}{{2F}^{2}I}}$$

where F is the Faraday number, εε^0^ is the permeability of the electric medium, T is the temperature, I is the ionic strength of 0.9% NaCl, and R is the gas constant [52].

## 4. Conclusions

Synthetic cathinones are one of the most dynamically developing groups of new psychoactive substances on the drug market. These substances appeared in Europe in 2005, and despite currently being under legal control, they continue to enjoy significant interest from users. Illegal laboratories producing cathinones are constantly being closed down, and large quantities are being confiscated. They cause numerous deaths and poisonings. Currently, a growing number of detailed studies on their effects on the human body are appearing in the literature. Synthetic cathinones primarily affect the central nervous system, but also cause other effects such as hallucinations, euphoria, agitation, and paranoia. To date, no studies have reported their effect on the electrical properties of membranes of morphotic blood; therefore, we investigated changes in zeta potential (surface charge density) as a function of the electrolyte pH in membranes modified with cathinones.

Comparing the curves obtained for mephedrone and clephedrone, common features are observed in some systems, suggesting that compounds with similar effects exhibit identical changes at the membrane surface. However, there are also sections of the curves that contradict this. Even comparing the results obtained for mephedrone and clephedrone, significant differences in the curves are visible, although these compounds have identical effects on the central nervous system. It can be seen that, in samples incubated for 24 h, the difference in the surface charge density obtained at the tested concentrations is smaller than in samples tested immediately after the substance was added. This may be because the added compound degraded in the whole blood sample within 24 h of the test. Based on the analysis of all curves, the results show that the tested substances interact more strongly with platelet membranes than with erythrocyte membranes. Based on the results obtained for mephedrone and clephedrone, it can be concluded that at the tested concentrations (170 ng/mL and 2700 ng/mL), they alter the zeta potential of the biological membranes of red blood cells and platelets. The studies conducted are preliminary; further experiments are needed to precisely determine the effects of cathinones on cell membranes. This research is essential because changes in biological membranes affect cell function, which, in turn, influences the function of the organ, which, in turn, influences the function of the entire organism. Therefore, results from in vitro studies on natural erythrocyte and platelet membranes can aid in interpreting and analyzing the equilibrium processes occurring at the membrane surface after ingestion of such substances.

## Figures and Tables

**Table 1:** Zeta potential, surface charge density and isoelectric point values for the erythrocyte membrane–mephedrone system immediately after isolation from whole blood and after 24 h. * Statistically significant difference (*p* ≤ 0.05) compared to control.

Examined System	Isoelectric Point	Surface Charge Density δ [10^−2^ C m^−2^]		Zeta Potential ξ [mV]	
		pH~2	pH~9	pH~2	pH~9
control	~3.9	1.04 ± 0.10	−1.66 ± 0.31	6.93± 0.60	−11.07 ± 1.13 *
mephedrone 170 ng/mL	~3.5	1.26 ± 0.13 *	−1.69 ± 0.38 *	8.26 ± 0.78 *	−11.27 ± 1.15 *
mephedrone 170 ng/mL after 24 h	~2.9	0.68 ± 0.09 *	−1.75 ± 0.54 *	4.53 ± 0.54 *	−11.67 ± 1.19 *
mephedrone 2700 ng/mL	~3.1	0.72 ± 0.09	−1.61 ± 0.54	4.82 ± 0.54 *	−10.73 ± 0.94 *
mephedrone 2700 ng/mL after 24 h	~3.4	0.39 ± 0.08 *	−1.65 ± 0.52 *	2.60 ± 0.48 *	−11.15 ± 1.12 *

**Table 2:** Zeta potential, surface charge density and isoelectric point values for the thrombocyte membrane–mephedrone system immediately after isolation from whole blood and after 24 h. * Statistically significant difference (*p* ≤ 0.05) compared to control.

Examined System	Isoelectric Point	Surface Charge Density δ [10^−2^ C m^−2^]		Zeta Potential ξ [mV]	
		pH~2	pH~9	pH~2	pH~9
control	~4.2	1.63 ± 0.21	−1.09 ± 0.09	10.87± 1.26	−7.27 ± 0.73
mephedrone 170 ng/mL	~5.1	1.70 ± 0.12 *	−1.66 ± 0.13 *	11.33 ± 1.37 *	−11.07 ± 1.11 *
mephedrone 170 ng/mL after 24 h	~4.8	1.29 ± 0.12 *	−1.71 ± 0.12 *	8.60 ± 0.86 *	−11.40 ± 1.10 *
mephedrone 2700 ng/mL	~5.0	1.47 ± 0.19 *	−1.33 ± 0.12 *	9.82 ± 0.95 *	−8.87 ± 0.84 *
mephedrone 2700 ng/mL after 24 h	~5.4	1.47 ± 0.09	−1.55 ± 0.07	9.80 ± 0.52 *	−10.33 ± 1.02 *

**Table 3:** Zeta potential, surface charge density and isoelectric point values for the erythrocyte membrane–clephedrone system immediately after isolation from whole blood and after 24 h. * Statistically significant difference (*p* ≤ 0.05) compared to control.

Examined System	Isoelectric Point	Surface Charge Density δ [10^−2^ C m^−2^]		Zeta Potential ξ [mV]	
		pH~2	pH~9	pH~2	pH~9
control	~3.9	1.04 ± 0.10	−1.66 ± 0.11	6.93± 0.67	−11.07 ± 0.73
clephedrone 170 ng/mL	~3.2	0.73 ± 0.12 *	−1.69 ± 0.09	4.87 ± 0.47 *	−11.27 ± 0.81 *
clephedrone 170 ng/mL after 24 h	~3.7	0.63 ± 0.09	−1.61 ± 0.11 *	4.20 ± 0.40	−10.73 ± 0.87 *
clephedrone 2700 ng/mL	~3.2	0.75 ± 0.11 *	−1.78 ± 0.09	4.82 ± 0.65 *	−11.86 ± 1.14 *
clephedrone 2700 ng/mL after 24 h	~3.5	0.53 ± 0.18 *	−1.71 ± 0.08	2.60 ± 0.52 *	−11.40 ± 1.02 *

**Table 4:** Zeta potential, surface charge density and isoelectric point values for the thrombocyte membrane–clephedrone system immediately after isolation from whole blood and after 24 h. * Statistically significant difference (*p* ≤ 0.05) compared to control.

Examined System	Isoelectric Point	Surface Charge Density δ [10^−2^ C m^−2^]		Zeta Potential ξ [mV]	
		pH~2	pH~9	pH~2	pH~9
control	~4.2	1.63 ± 0.21	−1.09 ± 0.09	10.83± 0.97	−7.27 ± 0.73
clephedrone 170 ng/mL	~4.0	1.71 ± 0.12 *	−1.66 ± 0.13 *	11.40 ± 1.07 *	−11.07 ± 0.91 *
clephedrone 170 ng/mL after 24 h	~4.2	1.22 ± 0.17 *	−1.69 ± 0.12 *	8.13 ± 0.82 *	−11.27 ± 0.98 *
clephedrone 2700 ng/mL	~5.1	1.91 ± 0.13 *	−1.72 ± 0.15 *	12.73 ± 1.25 *	−11.47 ± 1.14 *
clephedrone 2700 ng/mL after 24 h	~4.0	1.10 ± 0.13 *	−1.56 ± 0.12 *	7.33 ± 0.72 *	−10.41 ± 0.92 *

## Data Availability

The data presented in this study are available on request from the corresponding author.
